# Understanding the factors that influence CT utilization for mild traumatic brain injury in a low resource setting - a qualitative study using the Theoretical Domains Framework

**DOI:** 10.1016/j.afjem.2024.04.004

**Published:** 2024-05-06

**Authors:** Harriet Nalubega Kisembo, Richard Malumba, Henry Sematimba, Racheal Ankunda, Irene Dorothy Nalweyiso, Elsie-Kiguli Malwadde, Elizeus Rutebemberwa, Simon Kasasa, Dina Husseiny Salama, Michael Grace Kawooya

**Affiliations:** a Makerere University, College of Health Sciences, School of Medicine; b Department of Radiology, Mulago National Referral and Teaching Hospital, Kampala, Uganda; c Ernest cook Ultrasound Research and Education Institute, Mengo Hospital, Kampala, Uganda; d African Centre for Global Health and Social Transformation (ACHEST), Kampala, Uganda; e School of Public Health, Department of Health Policy & Management, Makerere University, Kampala, Uganda; f Department of Epidemiology & Biostatistics, School of Public Health, Makerere University, Kampala, Uganda; g National Center for Radiation Research and Technology, Cairo, Egypt

**Keywords:** Mild traumatic head injury, Computerized tomography utilization, Imaging referrers, Decision making, Theoretical Domain Framework, Low resource setting

## Abstract

**Introduction:**

In low resource settings (LRS), utilization of Computed Tomography scan (CTS) for mild traumatic brain injuries (mTBIs) presents unique challenges and considerations given the limited infrastructure, financial resources, and trained personnel. The Theoretical Domains Framework (TDF) offers a comprehensive theoretical lens to explore factors influencing the decision-making to order CTS for mTBI by imaging referrers (IRs).

**Objectives:**

The primary objective was to explore IRs’ beliefs about factors influencing CT utilization in mTBIs using TDF in Uganda.

Differences in the factors influencing CTS ordering behavior across specialties, levels of experience, and hospital category were also explored.

**Materials and Methods:**

In-depth semi-structured interviews guided by TDF were conducted among purposively selected IRs from 6 tertiary public and private hospitals with functional CTS services. A thematic analysis was performed with codes and emerging themes developed based on the TDF.

**Results:**

Eleven IRs including medical officers, non-neurosurgeon specialists and neurosurgeons aged on average 42 years (SD+/-12.3 years) participated.

Identified factors within *skills* domain involved IRs’ clinical assessment and decision-making abilities, while beliefs about *capabilities* and *consequences* encompassed their confidence in diagnostic abilities and perceptions of CTS risks and benefits. The *environmental context and resources* domain addressed the availability of CT scanners and financial constraints. The *knowledge* domain elicited IRs’ understanding of clinical guidelines and evidence-based practices while social influences considered peer influence and institutional culture. For *memory, attention & decision processes* domain, IRs adherence to guidelines and intentions to order CT scans were cited.

**Conclusion:**

Using TDF, IRs identified several factors believed to influence decision making to order CTS in mTBI in a LRS. The findings can inform stakeholders to develop targeted strategies and evidence-based interventions to optimize CT utilization in mTBI such as; educational programs, workflow modifications, decision support tools, and infrastructure improvements, among others.

**African relevance**

• The publication of these findings partly addresses the scarcity of literature on appropriateness of CT utilization from Africa
• Other countries in the region with a similar health infrastructure, disease profile, and health resources can use our experience and findings to develop their healthcare strategies to improve patients’ outcome.
• This paper underscores the value of the TDF in guiding research, policy, and practice in this area. Researchers in the region may reference it to conduct more theory-guided studies intended to change inappropriate clinical practice behaviors to develop tailored evidence-based interventions.

## Introduction

Mild Traumatic Brain Injury (mTBI) is defined as blunt injury to the head resulting in an unaffected or minimally altered level of consciousness with a Glasgow Coma scale (GCS) score of 13–15, and loss of consciousness for ≤15 min, or posttraumatic amnesia for ≤60 min, or both [1]. It is a global public health and economic challenge, with greatest burden being in in low-middle-income countries (LMICs) [2], [3], [4].

Computed tomography scan (CTS) is accurate in identifying intracranial injuries that may require immediate intervention or monitoring, such as hemorrhage or skull fractures [5]. This occurs in 7–12% of patients with mTBI, of which less than 1% will require surgery. In the rest of cases of mTBI, diagnosis can be confidently made based on clinical evaluation, including history-taking and physical examination without the immediate need for CTS [6], [7], [8].

Several studies have estimated the rate of unnecessary head CTS for mTBI [9], [10], [11], [12]. One study published in Cureus in 2023 found 22.6% of the 486 adult patients (>14 years) presenting to emergency departments with mTBI received unnecessary head CTS [11]

A recent systematic review that investigated CTS over-use in mTBI by Sarah M et al,[12] also found pooled rate of unnecessary CTS to be 27% [95% CI: 16–43; I2 = 99%]. The rate varied depending on various factors, including healthcare practices, clinical imaging guidelines (CIGs) availability and adherence, and individual patient characteristics.

Inappropriate use (over and under) of CTS procedures has negative consequences such as; increased radiation exposure, healthcare costs, resource strain, over diagnosis, false positive results, treatment delays, missed diagnoses associated with significant morbidity or mortality and legal and ethical concerns [13], [14], [15], [16].

The increased frequency of head injuries in Africa due to road traffic collisions (RTCs), coupled with limited access to diagnostic tools such as CTS, often concentrated in urban areas or major hospitals, and prohibitive costs, pose significant challenges for effective management and treatment of these injuries [17], [18], [19], [20].

Therefore, the reason and how healthcare workers decide whether a patient needs CTS is important and can have a big impact on imaging resources.

There are evidence-based decision support tools (EBDSTs) such as the Canadian CT head rules and clinical imaging guidelines (CIGs) to aid decision of identifying patients who are at low risk of intracranial injury and can safely be managed without imaging [21], [22], [23], [24]. These however were developed for high income countries (HICs) and there is a lack of evidence/validation for their use in low-medium income countries (LMICs).

Previous studies indicate several clinical- and nonclinical-interconnected factors influence emergency physicians' decision-making when ordering CTS for mTBI [25,26]. In the process of health care quality improvement, theoretical understanding of the factors involved in changing the behaviors of healthcare providers (HCPs) is important in the initial stages [27].

The Theoretical Domains Framework (TDF) is a comprehensive framework that integrates 33 psychological and behavior change theories to understand behavior change in healthcare settings.

It comprises 14 (originally 12) domains that can be used to identify factors influencing behavior [28,29]. These domains include knowledge, skills, beliefs about capabilities, beliefs about consequences, environmental context and resources, social influences, emotions, behavioral regulation, intentions, social/professional role and identity, optimism, reinforcement, goals, memory, attention, and decision processes.

This study therefore, aimed at applying the TDF to the context of CT utilization for mTBIs in a resource-limited setting (RLS), to understand factors believed by IRs to influence the decision to order CTS. We also aimed to identify differences in factors influencing CTS ordering behavior across specialties, levels of experience, and hospital category (public/private and urban /rural).

## Methods

### Study Design

This was a cross-section survey using in-depth semi-structured interviews (SSIs).

### Study setting

Six of the 22 hospitals (30%) with functional CT service in the Uganda participated. These included two public (tertiary referral hospital and university teaching hospitals), two private for-profit (PFP), and two private not-for-profits (PNFP). These Hospitals were purposefully selected based on geographical and CTS services representation in the country [30].

### Participant

Through a telephone interview, in-charge radiographers were requested to identify IRs who had referred patients aged ≤ 35 years for head CTS following trauma in the last six months from the requisition forms. The upper limit of 35 years was based on the attributable risk of cancer from low-dose radiation which plateaus beyond this age [31]. Other IRs was identified from attendance lists of continuous medical education (CME) activities for appropriateness of CTS held during another study period.

The selection of this diversity sample of IRs was based on the nature of practice behaviors in the study setting. Given the scarcity of neurosurgeons (NSs), the management of head injuries often falls on other HCPs, such as medical officers, emergency physicians, non-neurosurgeon specialists (nNSSs) or general practitioners. Different age groups, experience/ number of years of practice, NSs/nNSSs) and practice type/location involved in the management of mTBI were selected.

The identified IRs was invited to participate in the study via telephone calls. An information document outlining the purpose and requirements of the study was then sent to those who agreed to participate.

### Materials

An interview guide was used during SSIs followed the 12 different domains of the 2005 TDF adopted and adapted from previously published work in the same field by Bussieres et al [32]. (Appendix B). The guide was piloted with one IR and one radiologist to test face and content validity. This pilot was to ensure that questions adequately covered each theoretical domain and were relevant to the clinicians. Minor adjustments were made in the content and format and reduced the length of the questionnaire.

### Procedure

In-person SSIs were conducted at the IRs’ work place except for one interview which was held by telephone due to the remote location of the hospital Enrollment was concluded at the point when the range of ideas elicited from the interviews uncovered no new themes (thematic saturation) [33].

### Analysis

Interview transcripts were coded independently by two investigators (MD and MR) and disagreements were formally resolved at each step. The researchers had knowledge and experience in qualitative research and evidence-based medicine research methods and in-depth knowledge of the project. However, since none had used TDF before, they were guided by literature [28,29].

Thematic analysis was performed because the researcher would easily see the emerging themes and there was no need to condense the data and make further meanings. This involved identifying broad ideas, concepts, behaviors, and phrases. These were assigned with codes that were emerging; common themes were finally developed based on the TDF. The TDF phrases/fragments that were relevant to more than one domain were cross-referenced.

The interview guide was developed in the ‘Code Manager.’ *'Code Manager" refers to a feature or function within the ATLAS.TI software for qualitative data analysis that allows researchers to organize, manage and navigating the coding process.* Transcripts were transformed into Rich Text Formats which enabled the sets to be transferrable to the ATLAS.TI software that analyzed the data. Areas that needed further exploration were identified and reviewed; common themes were generated further and tailored to research objectives. Factors that were frequently mentioned were considered highly important. Quotations from the transcripts were cited to illustrate each domain and these required no editing for readability.

### Ethical approval

Ethical approval was obtained from the School of Medicine Research and Ethics Committee (SOMREC), REF: #REC REF 2017-118, and the National Council for Science and Technology (UNCST), REF: HS1313ES. Administrative clearance was also obtained from all the participating hospitals before the start of the study. All study procedures were in accordance with the ethical standards of SOMREC and/or UNCST and with the 1964 Helsinki Declaration.

## Results

A total of 18 IRs were invited to participate in the study. Seven IRs who did not respond after being contacted at least three times, or didn't consent or not based in the participating hospitals due to challenges of traceability were excluded. The 11 IRs were interviewed during one month (1^st^ to 31^st^ January 2020). The duration of the interviews ranged between 45 -60 minutes. At least one IR per participating hospital was recruited.

The average age of participants was 42 years (SD +/-) 12.3) and 18% (2/11) were females. Participants ranged from medical officers (MOs) with number of years in practice less than 5 years to specialists with up to 20 years of experience. Ten IRs were in full-time practice, which included clinical services and academics.

Participants’ socio-demographic data is summarized in Table 1 below:

**Table 1:** Demographic characteristics of 11 Imaging Referrers from selected Health facilities in Uganda

**Characteristic**	**Frequency**
**Gender** **Male** **Female** **Age**	**9** 2
25-34	4
35-44	4
45-54	1
55-64	2
**Cadre**	
Neurosurgeon	3
Medical officer	4
Physician	3
Pediatrician	1
**Type of Health facility**	
Public	2
*PNFP	2
*PFP	2
**Experience in years**	
1-5	4
6-10	0
11-15	2
16-20 21 and above	2 3
Employment	
Full time (> 25 hours /wk)	10
Part time (> 1-24 hours/wk)	1

⁎ PNFP = Private not for profit, PFP = Private for profit

**Figure 1:**
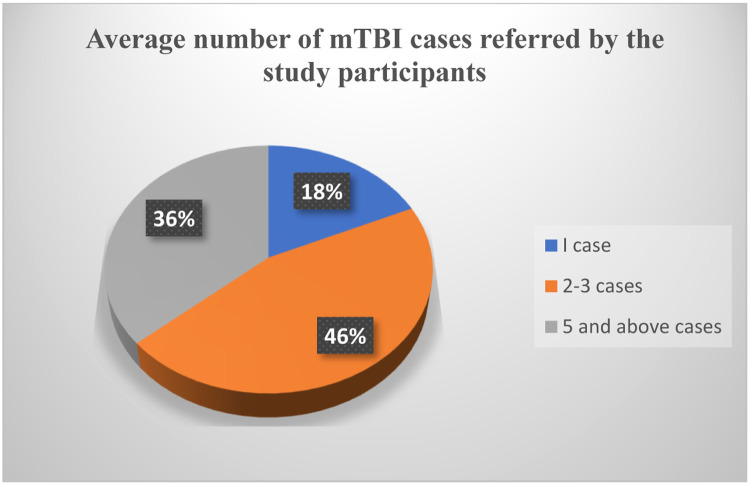
Number of weekly mTBI cases referred by the IRs

Out of the 11 IRs, five (46%) reported handling at least 2-3 cases of mTBI weekly as shown in Fig. 1 below:

The emerging themes were categorized into theoretical domains with illustrative quotes using the following identifiers: medical officer (MO), non-neurosurgeon specialist (nNSS) and neurosurgeon (NS) are summarized in Table 2 (appendix C)

The key factors perceived by IRs to influence CT ordering behaviors when managing mTBIs were categorized into mainly eight theoretical domains. These included skills, beliefs about capabilities, beliefs about consequences, environmental context & resources, social influence, knowledge, memory & decision processes, and social/professional role & identity.

In the domain of *skills*, good clinical acumen including history taking and physical examination of the central nervous system, assessing the severity of head injuries, and for red flags were cited consistently by all IRs to influence decision-making for ordering CTS:

*‘…I take history and find out the circumstances for the head injury; if I find out there was loss of consciousness, or seizure, is the patient weak or had amnesia? I assess for headache, vomiting, and bleeding in the ears and nose. I then examine the patient and look out for signs like fluid from the ears, changes in the pupils, periorbital changes, and discoloration around the ears and I ask the patient to move his limbs. If the patient is unconscious, I score him out of 15, and based on the answers to these queries, I will do or not do a Head CT. [NS3]*

In addition, good communication and counseling skills to manage patients’ anxiety and gain their trust in the doctors’ decisions were mentioned as important in reducing potential medical-legal issues if CTS is not done:

*‘…Communication skills are very important, to explain to the patient/caretaker why a CT scan is /is not necessary once you don't communicate well, the attendants may feel like you haven't done something about their patients. It also saves from medical legal issues.’ [MO1]*

Intolerance of uncertainty and receding fundamental clinical skills, replacing them with imaging technology was mentioned by one nNSS to increase CT ordering, especially among young doctors:

*‘…It's bad practice to do it the lazy way, where you do a CT scan and take it from there…… we emphasize clinical skills which have been there. Technology cannot replace good clinical skills. Young doctors’ threshold for doing CT scan is very low…’ [nNSS1]*

*Beliefs about capabilities* included IRs’ self-confidence in the ability to screen for red flags and be able to diagnose mTBI without CTS. This was acquired through training, experience generated over the years, exposure to very many cases of head injuries, support from colleagues, supervision and mentorship of juniors by the seniors as well as continuous professional development (CPD) activities.

On the other hand, concerns about missing intracranial injuries (domain of *consequences*), and the lack of an appropriate responsive nursing team, unreliable follow-up system (*environmental context & resources*), led to the overutilization of CTS:

‘*…I think we need a good follow-up system if CIGs are to work because most often those nations that developed the head CT rules have a good follow-up system in place. …if you are to look at the Canadian system, I have been in it before, in as much as we would send patients off without scan, they had a good follow-up, they had a GP to follow up, they had a pediatrician at home to follow up, family members are vigilant …, they have programs to alert caretakers on the tell-tale signs, they can access them on google, ——–but our protocols have no such programs……….’. (NS3)*

Regarding *beliefs about consequences*, utterances were heterogeneous. Seven IRs believed that managing mTBI without CTS reduced potential radiation risks, costs, misuse, and clogging of the imaging services:

*‘…The consequences are that we save money, and we spare the patient from radiation. We don't misuse and clog the service; we don't promote bad practice’. [nNSS2]*

However, all the neurosurgeons (NSs) believed, the risks and consequences of missing a significant brain injury outweighed the radiation a patient gets from a CTS:

*‘…I know that the big thing about you radiologist is to worry about radiation exposure, whereas us, the worry is about the benefits at that point…………. it's one CT versus no CT for a while’ [NS3]*

Regarding the domain of *environmental context and resources*, the key factors were mainly availability and accessibility of CT services. On-site CT equipment, affordability, a radiologist to interpret the image, turn-around time, and time when the request is made influenced the decision to order CTS:

*‘I think once you have something, there is an inherent bias to use it’ [nNSS3]*

Other factors such as work overload, understaffing, inadequate bed space for admission, and lack of a reliable follow-up system were also mentioned:

‘The *breakdown of clinical nursing care makes radiology an escape, by requesting unnecessary CT scans’ [nNSS1]*

*Social influences* such as the opinion of colleagues, patients, caretakers, expectations of receiving CTS, lack of tolerance for ambiguity and anticipated outcomes were mentioned to influence individual providers' decision-making for CT ordering practices:

*‘…Our colleagues, if they think I am over-investigating or under investigation, they will tell me. We discuss these cases all the time’ [nNSS4].*

Concerning the *domain of knowledge*, slightly less than 50% (5/11) of the IRs were aware of EBDSTs and only two were using protocols from the NSs:

‘*…Yes, the Brain-Trauma Foundation guidelines. These help me to classify head trauma and they are a bit detailed. The challenge is that they are developed for the first world countries and some recommendations aren't applicable here’ [NS3].*

However, all IRs highlighted the need to develop guidelines tailored to their local clinical setting to support decision-making when ordering for imaging.

**‘***…I can't name any guideline that I know but we need to use them for guidance. ‘[MO4]*

A secondary objective was to compare responses from NSs/nNSSs, experience (below 5 years /5 years and above), practice category (public/private), and location (rural vs urban).

Beliefs about most key domains and themes were generally similar across all IRs. However, the NSs perceived CTS as a more objective and reliable way for screening patients with mTBI for pathology to avoid delayed interventions and potential consequences, such as poor patient outcomes and medico-legal issues. On the other hand, nNSSs were more concerned about patient radiation safety and saving costs.

In the absence of EBDSTs, NSs relied more on their expertise and personal experiences to support decision-making to order CTS, while nNSSs and MOs depended on consultations and supervision from colleagues and seniors respectively. Although all IRs were in support of having tailored EBDSTs, only NSs had some knowledge and awareness of such tools, which they used inconsistently for supporting decision-making.

In hospital category (public /private), influencing factors were elicited mainly in the domains of *emotions, social influence, capabilities, and consequences*. Private patients and those with medical insurance coverage tended to be additional stressors to IRs compared to patients from public hospitals and those paying out-of-pocket respectively. To a lesser extent, defensive medicine, financial incentives, and self-referrals were mentioned by only two IRs from private hospitals. Rural compared to urban hospitals had poor access to neurosurgical care, CT services, support supervision, transportation, financial constraints, and cultural attitudes, which increased underutilization of CTS.

## Discussion

This study had IRs from different clinical practice settings (urban/rural, private/public), age groups, skills, and experience to give a diversity of complimentary views concerning the clinical practice behavior. The face-to-face interviews create a relationship between the interviewer and the respondent, which improves the depth of information obtained. The use of TDF to understand factors perceived by IRs to influence clinical decision-making when ordering CTS for mTBI, recruiting until saturation [34], two people conducting the interviews and the independent coding and analyzing the results added vigor to the methodology.

Eight key theoretical domains believed by IRs to influence a decision to order CTS in mTBI included: skills, beliefs about capabilities, environmental context, and resources, beliefs about consequences, memory, attention, decision processes, social influences, social/professional role & identity, and knowledge of EBDSTs.

The findings are consistent with the study by Tavender et al, which explored factors influencing doctors’ decisions to order CTS of the head in an emergency department[35].

Given the complexity of the central nervous system, HCPs required good clinical acumen, counseling, and communication skills to manage mTBI without CTS (skills domain). Senior IRs relied more on expertise and personal experiences gained over the years while the junior IRs relied more on support supervision from the senior doctors. The CPD activities and the frequent exposure to very many trauma cases due to high burden of road traffic collision in the study setting tended to enhance their confidence [17].

Rohacek et al, also found the absence of abnormalities in neurological examination in patients after mTBI to be a reliable indicator for omitting CT scanning [36]. Similarly, Melnick et al [14] and a systematic review by Borg et al [37], emphasized the importance of clinical skills in optimizing CT utilization in mTBI.

There is variability in the decision-making process regarding CT utilization for mTBI in many African countries due to unavailability of EBDSTs and standardized protocols for mTBI, with HCPs relying more on their clinical judgment [38].

Clinical imaging guidelines have been found to be effective in optimizing CT utilization in cases of mTBI by providing risk stratification criteria, reducing unnecessary scans, guiding clinical decision-making, standardizing practice, enhancing patient safety, facilitating education and training, and supporting quality improvement initiatives [ 23, 24].

Previous studies noted that cognitive overload and time constraints in emergency situations to lead to reflexive ordering of CTS instead of careful consideration based on guidelines [39,40].

While adopting /adapting clinical guidelines developed for HICs to LMICs may not be appropriate due to differences in healthcare infrastructure, resources, patient populations, and disease prevalence, they can provide valuable guidance, when specific needs, resources, and challenges of the local healthcare system are taken into account. Fostering collaborations and partnerships between healthcare organizations, academic institutions, and international stakeholders, such as IAEA, WHO, and ESR can support capacity-building efforts, optimize resource allocation and improve patient outcomes. An example of such collaboration is the African Regional Co-operative Agreement on Research, development, and training related to nuclear science and technology projects (RAF/9/064) aimed at improving justification of medical exposures [41].

The subject of radiation protection is barely taught in medical schools, except to those who advance as residents. There is neither curriculum nor policy to emphasize related courses of radiation safety. Some form of knowledge is obtained through self-reading and CME activities [19,38,42].

This is similar to studies done by Uri et al. in England where they found low levels of radiation awareness among IRs [43].

In order to strengthen standardized CT ordering practices, IRs’ knowledge and skills in mTBI assessment and CT utilization could be enhanced through CPD programs and educational initiatives focusing on evidence-based practices, risk assessment strategies, and the appropriate use of CT imaging.

### Study limitations

The limitations of this study are that the identified factors were views of the interviewed IRs, and may not provide the actual evidence of influencing factors on the clinical practice behavior [34]. In addition, the factors were identified using a cross-sectional study design which may change over time. It is possible more factors would surface if we were to interview other stakeholders like patients, caretakers, radiologists, regulators, administrators, and proprietors other than only IRs.

## Conclusion

Using the TDF, we have systematically explored the multidimensional factors surrounding the decision-making process for CT utilization in a LRS.

The findings may inform stakeholders to develop contextualized targeted strategies and EBIs to optimize resource allocation, improve clinical decision-making, and enhance patient-centered outcomes for mTBI. These may include improving access to imaging services, reducing costs, strengthening healthcare infrastructure, enhancing provider training, and developing context-specific CIGs and protocols for managing mTBI.

### Dissemination of results

Results from this study will be shared with imaging referrers from the participating hospitals through routine CMEs and professional annual conferences. The research findings will also be summarized in clear user-friendly language to ensure that the information is accessible to a public audience in form of posters and policy briefs. The local communities, patient advocacy groups, and community-based organizations will be engaged through educational workshops, public forums, and informational materials.

## Authors’ contribution

Authors contributed as follows to the conception or design of the work; the acquisition, analysis, or interpretation of data for the work; and drafting the work or revising it critically for important intellectual content: HNK 40 %, RM 14 %, HS 6%, and RA, RN, INK, EKM, ER, SK, DH and MGK each contributed 5%. All authors approved the version to be published and agreed to be accountable for all aspects of the work.

## Declaration of competing interest

All authors declared no conflicts of interest

## Appendix Supplementary materials

Image, application 1

Image, application 2
