# Supplementary material for: Understanding the factors that influence CT utilization for mild traumatic brain injury in a low resource setting - a qualitative study using the Theoretical Domains Framework
Source: Afr J Emerg Med. 2024 May 6;14(2):103–8. doi: 10.1016/j.afjem.2024.04.004 (PMC11096711; doi:10.1016/j.afjem.2024.04.004)
Supplement: Supplementary file 1 [file mmc1.docx]

**Appendix B: Qualitative research study (QRS) : Script for the Introduction of the in-depth Interviews**

Adopted and adapted from Bussières AE et al [32].

Introduction by interviewer

‘My name is …., and this is _________. I would first like to thank you for accepting to take part to this interview. The general aim of the interview is to enable us to understand how you manage patients presenting with mTBI and what are the perceived barriers and facilitators of inappropriate CT requisitions in your own clinic. This interview is based on multiple related theories to help us understand the care provider’s practice, so some questions may sound similar.

I would like to invite you to briefly introduce him/her and tell us in what setting you practice, and the type of work in relation to healthcare

“I’m just going to read you a brief description of what we are doing today, which we are reading to everyone we interview.”

“Our goal today is to talk about how you manage new patients presenting with mTBI. Also, we are interested in knowing what problems if any, you may see in implementing the imaging guidelines in your daily practice. We are here to listen to your experiences to help us understand these two issues better”.

“You should have received an e-mail containing the invitation letter with links to websites for you to review key recommendations in the ESR iGuide full guidelines. We are interested in knowing your thoughts on this. If you didn’t have a chance to review it, please take a few minutes to do so now.”

There are a few things I’d like to touch on before we get started:

1. Any discussion that occurs in this room stays in this room. All of the information that is shared with us is confidential. You can feel free to say what you like related to the topic at hand. 2. Your participation in this interview is voluntary. You can choose not to answer any of the questions if you don’t want to, and you are free to leave at any time with no consequences, including without loss of incentive.
2. There are no right or wrong answers. We are interested in understanding perspectives about managing MTBI and using CT SCAN, so please answer frankly.
3. You shouldn’t feel that you have to agree with everyone else if that’s not how you feel. There are ______ people that will be interviewed, so we expect that people will have different views. And we must learn from all of the views that are represented here.
4. We want you to feel comfortable saying good things as well as critical things. We’re not here to promote a particular way of thinking about how to manage patients or about using CT scan guidelines. We just want to understand how clinicians such as your selves make sense of it.
5. We ask that you talk loudly so that we can be sure to hear everyone’s views and get them on tape.
6. I would like to remind you that this interview will be taped to preserve the maximum information you will share with us. As mentioned in the informed consent, once the transcription is completed your name will no longer appear on the documents. 8. (Identify note taker) Though we have someone taking notes of what is said in the interview, we also plan to record our discussion because it is important that we keep track of everything that is said during the interview. This will help us get the most accurate account of what is said and this will also help the note-taker in case the conversation speeds up and they are not able to write everything down. Is it okay with you that we record the session?
7. When you first say something, please say your name so that the person transcribing the tape will know who is talking.
8. If you have a cell phone or pager, I would ask that you please make sure this is turned off before we start.
9. And lastly, the session will take between 30 to 60 minutes. Please let me know if you need to take a break at any time. If you wish to find out more about this study, please contact (name/telephone number). Are you ready to start?

**Interview guide for IDI /IR (Elicited beliefs within TDF domains )**

Date of the interview……………………………….

Health Facility …………………………………………..

Introduction

I’d like to start by asking you to state your name slowly for the benefit of the person who will be transcribing the interview………………………………………………………………..

We understand management strategies may vary somewhat depending on whether patients have medical insurance or not. Because we are interested in getting a wide range of information, we would appreciate it if you could share your views for both insurance and non-insurance patients. This will make our findings more relevant to the routine health practices in our setting. The behavior of interest for today’s discussion is « managing new patients with minor traumatic head injury (MTBI) without taking CT scan. I’m going to keep emphasizing this throughout the interview.

1. Please state your Age --------------(in years)
2. Gender: 1= Male  2= Female 
3. Profession
   - 1. Radiologist
     2. Medical officer
     3. Specialist
     4. Others, please specify …………………………………………
4. If the profession is c, what is your specialty?.................................................
5. Approximately how many years have been practicing?.................................................
6. How many patient contact hours do you have per week in this facility?
   1. Full time (over 25 hours/wk)
   2. Part time (between 1-24 hours/wk)

**SECTION 2**

I would like to ask you to tell me in general how you might manage a new patient with MTBI in your clinic. By that I mean your usual routine once the patient is with you in the examining room, the questions you ask, and the exam procedures you do to determine if a CT scan is needed.

**Prompts**: What do you feel you do well? What do you feel you could do better? Thank you.

Now for the rest of the interview, I have some slightly more specific questions. Some may seem repetitive, but please bear with me as the questions are derived from multiple theories on human behavior and we are trying to identify which theory best applies in this area. I may also ask for clarification during the interview using probes such as:

‘What do you mean’; ‘Would you explain that’; ‘What were you thinking at the time’; ‘Take me through the experience’; ‘What skills are required to do so?’; ‘How and why do you use it?’

For this study, Mild TBI is defined as a blunt injury to the head that results in a normal or minimally altered level of consciousness in the patient at presentation to the emergency department i.e., a Glasgow Coma scale (GCS) score of 13–15, and loss of consciousness for ≤15 min, or posttraumatic amnesia for ≤60 min, or both. (151)(Carroll et al. 2004).

**Nature of the behaviors**

1. In your practice, how often do you come across new patients with MTBI?
2. What percentage of those patients do you do CT SCANS?

***prompts:*** do you systematically screen your patients for the presence of red flags?, do you assess the patient's motivation of undergoing CT SCAN or no CT SCAN and if so, how?, What do you usually say to patients who ask to undergo CT SCAN but where you find it is not clinically warrant? Do you follow up to monitor patients’ progress after treating them without a CT scan? What is the outcome usually?

**Skills**

1. How easy or difficult is it to manage a new patient with MTBI without taking CT SCAN? Why?

***Prompts:*** patient’s compliance, lack of training, complexity of cessation guidelines, lack of

**Counseling skills**

1. What skills are required to) manage a new patient with MTBI without ordering CT SCAN?

***Prompt***: how easy or difficult those skills are.

1. How much expertise or experience do you think one needs to have to manage MTBI in a new patient without taking CT SCAN?

***Prompt***: history taking, physician exam

**Beliefs about capabilities**

1. How confident are you that you can manage MTBI in a new patient without taking CT SCAN despite any difficulty?
2. What problems/difficulties do you think you might encounter in managing MTBI without CT SCAN?
3. What could help you overcome these problems/difficulties?

***Prompts:*** additional training, communication techniques, continuing education, educational material, online information. Please elaborate on whether or not you think communication skills are important for managing patients without CT SCAN. Why is that?)

**Motivation and goals**

1. How important do you feel it is (i.e. priority) to manage a new patient with MTBI without taking CT SCAN? (about other tasks like history taking and examination or others)
2. How does this differ between insurance and non-insurance patients?

***Prompts***: Are there any incentives that motivate you to manage patients with MTBI without taking CT SCAN? - Recommendations from UCG, from Regulatory body, colleagues, the medical community, or patients themselves

1. Would there be anything else that you want to do or achieve that might interfere with your practice with regard to managing a new patient with MTBI without taking CT SCAN?
2. Are there any incentives that motivate you to manage a patient with MTBI without taking CT SCAN?

***Prompt***: goals within yourself? External?

1. Do you set goals for yourself or your practice with regard to managing a new patient with MTBI without taking CT SCAN?

***Prompt:*** goals within yourself? External?

**Beliefs about consequences**

1. What are the benefits of managing a new patient with MTBI without taking CT SCAN?

***Prompt:*** to self, or patients i.e. reducing ionizing radiation exposure & costs? profession, healthcare organization

1. Do you feel there are potential harms or disadvantages in managing patients with mTBI without CT SCAN?

***Prompts:*** 1) accurate prognosis; 2) patient preference and satisfaction; 3) medico

1. Do you feel that the benefits of managing a new patient with mTBI without taking CT SCAN outweigh the costs? Why or why not?
2. Is there any incentive/disincentive that you can think of that influence whether or not you manage?

a new patient with mTBI without taking CT SCAN?

Environmental context and resources

1. What aspects of your environment (physical vs. resource factors) influence whether or not you are able to order CT SCAN for patients with mTBI?

***Prompt***: resources available to help you manage MTBI patients without taking

CT SCAN (information pamphlet or posters to inform patients about potential risks of ionizing radiation exposure)?

1. Do you think having onsite radiology equipment influences the decision to SCAN new patients with mTBI?

**Social influences**

1. How might the views/opinions of others (colleagues, patients, professional groups) influence your decision to SCAN patients with MTBI?

***Prompt:*** anyone else; in what circumstances

1. Do organizations such as INSURANCE influence whether or not you order CT scans for new patient with mTBI?

**Emotion**

1. Do patient emotions/apparent distress ever affect whether or not you order CT scan for patients with mTBI?
2. Does managing patient with mTBI without taking CT scan evoke an emotional response in you?

***Prompt:*** Do your own emotions ever affect your decision to manage patients with MTBI without taking CT scan?

***Prompts***: fear of missing a significant pathology

**Knowledge**

1. We have talked about some of the evidence; I’d also like to find out about your knowledge and use of

Guidelines:

1. Do you use any guidelines to help you make informed decision about when to CT SCAN for patients with head injury?

***Prompts***: How do you use it? Why do you use it? What do you think of it?

1. How do you use the guidelines? (i.e. what do you actually, physically do? Do you ever read the guidelines to check if a behavior you performed was guideline-compliant?)
2. What other evidence are you aware of?
3. How well do you think you understand the evidence surrounding optimal use of CT SCAN for MTBI?
4. What are your thoughts about the recommendations proposed by the ESR IGUIDE?

***Prompts:*** Do you believe it to be evidence-based? how did you use it? When did you use it?

What do you think of it?

1. Do you agree with the guidelines? i.e. are the guidelines representatives of the evidence (quality, appropriateness)?
2. What kind of additional information would most likely influence your changing your clinical management?

***Prompts:*** Random clinical trials, Systematic reviews, discussion with colleagues, conferences/seminars

**Memory, attention, and decision processes**

1. What thought process might guide your decision to manage a new patient with MTBI without taking CT SCAN?

***Prompt:*** What goes through your mind?

***Prompt:*** Is this something you would have to think a lot about?

1. Is managing patients with MTBI without taking CT SCAN something you would usually do?
2. In what situation, if any, might it be difficult to think of an alternative to using CT SCAN?
3. What rules of thumb do you use to reach a decision, if any?

***Prompts:*** red flags, decision rules, guidelines…)

***Social/professional role and identity***

1. Do you think it is an appropriate part of your job to manage new patients with MTBI without taking CT SCAN?

***Prompts:*** What are they and why? Do your colleagues generally agree with you on this issue?

1. Do you sometimes feel constrained by guidelines? What about protocols?
2. Is there anything else about your professional role that influences you managing patients without referring for CT SCAN? (i.e. Consensus in the clinical profession)
3. Do you tend to practice using CT scan-driven techniques to establish a treatment protocol?

***Prompts:*** What are they and why?

***Behavioral regulation***

1. If you’re thinking about changing your practice to manage new patients with MTBI without taking CT SCAN, how would you do it?

***prompt***: self-change, practice level change, training, education

1. What might you do in order to reduce the likelihood of needing CT SCAN?
2. Are there procedures or ways of working that might encourage you to manage a new patient with MTBI without taking CT SCAN?
3. If you decide to manage MTBI without taking CT SCAN, how confident are you that your associates can carry this out?
4. The evidence from research suggests that CT SCANS - are not useful for MTBI.

- With that in mind, in terms of using less CT SCANS:
- What might need to be done differently?
- What would you do differently?
- Who needs to do what differently, when, where, how, how often and with whom?

***That’s all the questions I had for you today. Has anything else occurred to you about this topic that we haven’t asked about?***

Overall, what were your thoughts about the interview?
