# Supplementary material for: Understanding the factors that influence CT utilization for mild traumatic brain injury in a low resource setting - a qualitative study using the Theoretical Domains Framework
Source: Afr J Emerg Med. 2024 May 6;14(2):103–8. doi: 10.1016/j.afjem.2024.04.004 (PMC11096711; doi:10.1016/j.afjem.2024.04.004)
Supplement: Supplementary file 2 [file mmc2.docx]

Appendix C

Table 2: Elicited beliefs of imaging referrers grouped by Theoretical Domains Framework influencing CT utilization in mTBI

| TDF domains | Themes | Specific beliefs | Representative quote |
| --- | --- | --- | --- |
|  |  |  |  |
| Nature of the behaviors | CT scan utilization | CT scan is performed in   - Majority of new patients with mTBI - Unknown patients - Unknown mechanism of injury - CTS not routinely done but, I monitor patients | *‘About 80%/90% of new patients with mTBI I do CTS’ [NS1]*  *‘We scan most of patients brought in as unknown and those we don’t know mechanism of injury’[MO2]*  *……. About 25-40% of new patients with mTBI I do CTS, the rest I follow up[nNSS2]* |
| Skills | Patients’ compliance, lack of training, counseling skills,  expertise /experience (history taking, physical exam) | 1. **Barriers**  - Inadequate experience &expertise to examine the central nervous system without missing any red flag - Inadequate training   to screen and determine if imaging is necessary   - Inadequate communication skills to explain to the patient the diagnosis - Inadequate counseling skills to convince a patient that a CTS is not needed - IRs and patient’s belief that CTS is useful for management of head injury | *‘…If there are gaps in history or mechanism of injury, cases of intoxication or unknown time for loss of consciousness especially if it is an accident unknown victim, it becomes harder to manage a mTBI case…’ [NS2]* |
|  |  | 1. **Facilitators**  - Experience gained from exposure to many traumatology patients - Good clinical acumen, history taking and clinical examination - Good communication and counseling skills to educate and reassure the patient that a CT scan is not necessary | *‘…It's very important, because when you've sent a patient home and you don't explain the result to come back, they don't know why they should come back. …’ [ NS3]*  *‘I take history and find out the circumstances for the head injury; if find out there was loss of consciousness, seizure, is the patient weak or had amnesia? I assess for headache, vomiting, bleeding in ears and nose. I then examine the patient and look out for signs like fluid from ears, changes in pupils, periorbital changes, discoloration around ears and I ask the patient to move his limbs. If the patient is unconscious, I score him out of 15 and based on the answers to these queries, I will do or not do a Head CT.’ [NS3]’*  *‘There are very many skills but I use the Glasgow Comma scale (GCS) and 'Alert', 'Voice', 'Pain’. Others are 'Unresponsive'(AVPU) although it isn’t clear and may not give much information. I also employ clinical acumen, history taking and clinical assessment skills…’ [ MO2]*  *‘…Communication and counseling skills are very important. Once you don’t communicate well, the attendants may feel like you haven’t done something about their patient. It also saves from medical legal issues.’ [MO1]* |
| Beliefs about capabilities | Confidence in eliciting red flags  Additional training, communication techniques, continuous medical education, educational materials, online information, communication skills | 1. **Barriers**  - Replacement of clinical skills with technology - Intolerance to uncertainty - Lack of CIGs/ Protocols |  |
|  |  | 1. **Facilitators**  - Experience and expertise - Supervision of Juniors by seniors - Appropriate Training, CMEs, access to educational materials, online information, material - Good communication skills | *‘…Sometimes management of mTBI cases is difficult but if one has been exposed to such cases long enough, 2 to 3 years’ experience is good exposure for the needed expertise…’ [MO4]*  *..’*  *‘…I am 95% confident that I can manage a* new patient with *mTBI… without CTS. I have interacted with many cases and I have had a chance to practice in a setting without a CTS. This has equipped me to handle those cases…’ [ MO3]*  *‘I don’t practice CTS driven practices but clinical acumen driven. CTS compliments my management but not influence it[MO2],* |
| Motivation and goals | Intrinsic motivations  Incentives, guidelines, colleagues, medical community, patients , regulatory body  Goals within /external | 1. **Barriers**  - Lack of CIGs /Protocols - Replacing clinical skills with technology | *‘Not knowing what you are dealing with, thinking its mild only later to discover its severe mTBI…..’ [NS1]*  ‘…*Its bad practice to do it the lazy way, where you do a CTS and take it from there…… we emphasize clinical skills which have been there. Technology cannot replace good clinical skills. Young doctors’ threshold for doing CTS is very low’ [ nNSS1]* |
|  |  | 2.**Facilitators**   - Awareness of effects of radiation exposures - good clinical evidence based practices - Patient centered care - Past experience and training. | *‘The cost of subjecting a patient to radiation when they don’t need it is key in my decision making’[ nNSS2]*  ‘*I have personally set my own criteria from past experience how to manage of mTBI without a CT scan with good recovery*  *[ nNSS2]* |
| Beliefs about consequences | Potential harms/disadvantages, accurate diagnosis, patients preference and satisfaction, medico legal issues  Benefits (self, patients, ie reducing ionizing radiation& costs) | 1. **Barriers**  - Pressure from patients/caretakers - Uncertainty of clinical diagnosis - Fear of missing life-threatening bleeds - Fear of litigation - Gaps in the monitoring and following up system of patients who have not performed a CT scan | *‘…I think you can’t manage a patient properly if the patient needs a CTS and you don’t do it. There is a risk of mismanagement leading to medical legal issues…’ [NS1]*  *‘…I know that the big thing about you, radiologist is to worry about radiation exposure, whereas us, the worry is about the benefits at that point…. it’s one CTS versus no CTS for a while’ [NS3]* |
|  |  | 1. **Facilitators**  - Patients confidence in the HCP - Awareness of risks of radiation by both the patient and the HCPs - consequences of unwarranted imaging | *‘…It is very important to manage such patients without CTS scans. Technology is there but it shouldn’t be misused. CTS emits radiation which is dangerous…’ [nNSS3]*  *‘Not doing CTS in mTBI saves doctors time, for the patient saves finances and exposure to radiation’ [nNSS2]* |
|  |  |  | *‘I get trust from my patients. If I don’t request for a CTS and it’s the correct decision’[MO2]*  ‘…*The consequences are that we save money, and we spare the patient from radiation. We don’t misuse and clog the service; we don’t promote bad practice…’ [nNSS1*] |
| Environmental context & resources | Environment (physical/resources), resources (information pamphlet, posters, signages ,  Onsite imaging equipment | 1. **Barriers**  - Having onsite CT equipment - Self-presentation - Resources including availability, affordability finances/costs, CTS interpretation, turnaround time - Challenges of following up of patients when discharged without a CTS - The time of the day (day / night) when patient present - Lack of patient information concerning red flags, radiation risks - Unavailable /not aware / lack of adherence to institutions guidelines and protocols - Workload and understaffing - Lack of information technology eg electronic medical records /radiology information system to integrate decision support tools - Inadequate expertise/neurosurgeons | *‘…The availability of the CT machine in-house, quick interpretation of the image, and the turnaround time for results influence me to use Head CTS. if you know that you are going to get your CTS results immediately, you request for it… [nNSS2]*  *‘I think once you have something, there is an inherent bias to use it’[nNSS3]*  *‘If I don’t have a good nursing team, I would rather do a CT scan’ [nNSS4]* |
|  |  | 1. **Facilitators**  - A good nursing team - Good clinical practice - Lack of onsite CTS | *‘...because there are patients who cannot afford CT scans., we usually discharge, especially the mildly head injured, we see them almost the next Monday to make sure that they are ok…’ [ NS2]*  *‘… if I have a good responsive nursing team, i can use watch and wait approach[nNSS1]* |
| Social influences | Views /opinions of others  (colleagues, patients, professional groups); influence of  Organization(insurance ) and new literature | 1. Barriers  - Views /opinions of others colleague/ seniors - The patients or caretakers’ emotions - Insurance companies - Patients expect to be investigated with imaging | *‘… if you get a case and discuss it with a consultant and he tells you to do a CTS, you may not go against it…’’ [ MO1]*  *‘…Despite the fact that I order for CTS with indication, the insurance people have access and just want to spend the money on their package. If not advised well, they will just ask for CTS yet they don’t need them….’ [nNSS1]*    *‘…I am senior enough; I take my decision. …’ [[nNSS2]*  *‘…If the patient or relatives insist, it’s hard to say no to request for a CTS. At times we give in to pressure from relatives and if the CT is in-house, it also speeds up the requisition of the Head CTS…’. [MO4]* |
|  |  | 1. **Facilitators**  - Previous experience and seniority. - Teamwork and multi-disciplinary approach - Dialogue and consultation with radiologist | *‘…Our colleagues, if they think I am over-investigating or under investigation, will tell me. We discuss these cases all the time…’ [nNSS4]*  *‘Dialogue and consultation with radiologist all the times’[nNSS1]* |
| Emotions | Own / patients emotions pathology | 1. Barriers  - Fear of missing a significant - Patient emotions | *‘…Yeah, I think this is tricky. Sometimes you come across situations when a patient is hysterical and worried. If the benefit outweighs the risks, I ask for it…’ (MO1).*  *‘Anxiety of doctors discharging a patient not knowing what’s happening and not sure the patient/caretaker will be able to recognize the red flags and come back for management’(NS3)* |
|  |  | 1. Facilitators  - Ability to judge the patient’s emotions from the symptoms /emotions | *‘…You must judge the patient's emotions from the symptoms. The emotions can influence you…’ [MO1]*  *.*  *‘…Emotions do less because my role as a Dr. is to show the patients or attendants why it is needed…’ [MO3].* |
| Knowledge | Awareness, knowledge and use of any guidelines  Quality/appropriateness | 1. **Barriers**  - Unavailable/un awareness/adherence to institutional guidelines/protocols - Unsuitable guidelines from developed countries - Limited knowledge concerning radiation risks - Misconception by patients concerning advanced imaging technology - Lack of information for patients/caretakers concerning red flags, risks of radiation | *‘Currently we don’t have any formal written guidelines in our setting ’ [ MO3]*  *‘…Yes, the Brain-Trauma Foundation guidelines. These help me to classify head trauma and they are a bit detailed. The challenge is that they are developed for the first world countries and some recommendations aren’t applicable here…’ [ NS3]* |
|  |  | 1. **Facilitators**  - Training and knowledge from textbooks - Experience - Institution guidelines /protocols from the neurosurgeons | *‘…No, I don’t know of any guidelines. Maybe the textbooks I read…’ [ MO2]*  ‘ *I draw knowledge from my training and clinical acumen to avoid unnecessary CTS’ [ MO2]* |
| Memory, attention, decision processes | Thought process: what goes through the mind  Rules of thumb (red flags, decision rules, guidelines) | 1. **Barriers**  - Lack of institutional imaging guidelines for mTBI | *‘We don’t have any protocols, we use clinical acumen’ [ MO1]* |
|  |  | 1. **Facilitators**  - Guidelines from neurosurgeons - Consultations from experts/neurosurgeons - Clinical sessions with juniors by neurosurgeons eg post admission rounds. | *‘Guidelines help u to support decision making and reduce medico-legal issues’[ NS]*  *‘We usually consult neurosurgeons for new challenging patients’[ nNSS2]*  *‘We use the guidelines. We have them on the phones, guidelines from the neurosurgeons’ [MO4]* |
| Social/professional role & identity | Professional role; professional  norms; professional agreement  constrained by guidelines /protocols  consensus in the clinical profession  CT scan driven techniques | 1. Barriers  - Lack of protocols and CIGs - Inadequate expertise - Inadequate support supervision of juniors | *‘For me It’s One man’s show, no interaction with colleagues’ [NS2]* |
|  |  | 1. Facilitators  - induction & orientation sessions for interns, new medical officers. - Support supervision, consultation and teamwork. - Continuous professional development sessions - educating the local population on the use of CTS - We need to put in place a protocol, well written, and users trained - Seniors as champions /potential opinion leader | *.*   - *‘Seniors as champions. Juniors usually learn from seniors’[MO1]* - *‘Many patients may not have the resources to do a CTS and so you use the window period to collect the money’[nNSS2]* |
| Behavior regulation | Practice behaviors change; self-change, practice level change, training , education evidence based intention | 1. Barriers  - Lack of CIGs - Lack of standardized clinical practice - Unaware of the institution protocols/guidelines  1. Facilitators  - Good clinical acumen - Refresher training courses/ CME - Clinical-radiology dialogues - Support supervision & mentorship | ‘*We need to set our own guidelines because we have a large patient load, they should be tailored to our setting’* *.[NS1]* |

Abbreviation

Computed tomography scan (CTS), mild traumatic brain injury (mTBI), clinical imaging guidelines (CIGs), Continuous medical education (CME), Health care providers (HCPs), medical officer (MO), non-neurosurgeon specialist (nNSS), neurosurgeon (NS)
